# Evaluation of Anticoagulation Control among Patients Taking Warfarin in University of Gondar Hospital, Northwest Ethiopia

**DOI:** 10.1155/2021/7530997

**Published:** 2021-12-21

**Authors:** Zelalem Liyew, Abilo Tadesse, Nebiyu Bekele, Tewodros Tsegaye

**Affiliations:** Department of Internal Medicine, School of Medicine, College of Medicine and Health Sciences, University of Gondar, Gondar, Ethiopia

## Abstract

**Introduction:**

Warfarin is a widely used oral anticoagulant in clinical practice. It has variable intraindividual and interindividual dose response and a narrow therapeutic index. Therefore, it requires frequent and regular international normalized ratio (INR) determination to maintain the INR within the therapeutic range. The study evaluated parameters of anticoagulation control among patients on warfarin.

**Methods:**

A cross-sectional study was conducted at University of Gondar hospital. A consecutive sampling method was used to recruit study subjects. The anticoagulation control was evaluated by determining the proportion of desired INRs and the proportion of time spent in the therapeutic range (TTR). Logistic regression analysis was used to identify associated factors with adequate TTR. A *P* value <0.05 was used to declare significant association.

**Result:**

A total of 338 study subjects were included in the study. The mean age of patients was 48.8 (SD = 16.4) years. Atrial fibrillation was the commonest indication for warfarin therapy. One-third (33%) of study subjects achieved the desired INRs of 2.0–3.0, while about one-tenth (13%) of patients attained good INR control (TTR ≥ 65%). Multivariate logistic regression analysis revealed no significant association of sociodemographic and clinical characteristics with good TTR outcome.

**Conclusion:**

The level of anticoagulation control with warfarin among study subjects was very low. The authors recommend to implement a validated warfarin-dose titration protocol and to establish anticoagulation clinics to mitigate the low anticoagulation level.

## 1. Introduction

Warfarin is the widely prescribed oral anticoagulant in clinical practice. It is used to prevent and treat arterial emboli in patients with atrial fibrillation and treat deep venous thrombosis [1–4]. Warfarin is a challenging drug which has variable intraindividual and interindividual dose response and a narrow therapeutic index. Therefore, it requires frequent and regular INR monitoring to maintain the INR within the therapeutic range [3, 4]. The proportion of INRs within the range of 2.0–3.0 (INR = 2.5–3.5 in prosthetic heart valves) determines the desired INR value. Therapeutic efficacy of warfarin will be reduced when INR value <2.0 and intrinsically lost when INR value <1.5 [3–7]. The proportion of time spent in the therapeutic INR range (TTR) evaluates the quality of anticoagulation control [3–7]. TTR ≥ 65% declares “good” INR control. TTR < 65% confirms reduced warfarin efficacy and increased risk of thromboembolic events or bleeding episodes. TTR < 40% notifies loss of survival benefit with warfarin use [7–11]. Sub-Saharan African studies documented that proportions of desired INR value = 2.0–3.0 and TTR ≥ 65% were 30–40% and 15–25%, respectively [12–16]. Global studies documented that old age, obese individuals, other concomitant drugs intake, excessive alcohol intake, and renal or hepatic dysfunctions were among the listed causes of poor TTR outcome (TTR < 65%) [8–11, 17–21]. Despite the wide availability of warfarin as an oral anticoagulant, information on its level of anticoagulation control is scarce in Ethiopia. This study aimed to determine the magnitude of proportion of INRs within the desired range, proportion of time spent in the therapeutic INR range, and factors associated with quality of anticoagulation outcomes in patients taking warfarin in a hospital setting, in Northwest Ethiopia.

## 2. Methods

### 2.1. Study Setting

A hospital-based cross-sectional study was conducted between November 1, 2019, and October 31, 2020, at Cardiology and Hematology Clinics, University of Gondar hospital, Northwest Ethiopia. The Cardiology and Hematology units provide healthcare services for outpatients who were followed up at the clinics. Both clinics were run by internists, medical residents, and respective unit nurses. Patients were admitted every one to three months based on their severity of illness.

### 2.2. Study Population and Study Subjects

Patients older than 18 years and who were on warfarin with follow-up at Cardiology and Hematology Clinics, University of Gondar hospital, during the study period were the study population.

### 2.3. Inclusion Criteria

Patients aged 18 years old and above, who were on warfarin for at least 6 months, had at least 6 INR determinations, and had each consecutive INR determination less than 2 months apart were included in the study.

### 2.4. Exclusion Criteria

Patients who did not give consent to the study, refused to be included in the study, and had incomplete data were excluded from the study.

### 2.5. Study Variables

Dependent variables included the proportion of time spent in the therapeutic INR range (TTR).

Independent variables included the following: (1) sociodemographic characteristics including age, gender, occupation, marital status, educational level, income level, residence, and religion; (2) clinical characteristics including indication for warfarin, warfarin dosage, concomitant drugs intake (antiplatelets, statins, antihypertensive drugs, antithyroid drugs, antiretroviral drugs), adherence to warfarin, INR monitoring interval, co-existing comorbidities, body mass index, and alcohol intake.

### 2.6. Sample Size and Sampling Procedure

The sample size was calculated using a single population proportion formula with the assumption of 95% confidence level and 5% margin of error and taking 30% for TTR in the sub-Saharan African region [12]. A consecutive sampling method was used to recruit 338 study subjects.

### 2.7. Data Collection Instrument and Procedures

Data were collected through an investigator-administered predesigned questionnaire. The questionnaire was prepared in English and translated into local language (Amharic) for data collection and then retranslated back to English while maintaining its consistency. The questionnaire had been pretested on 34 patients in a similar setup before the actual data collection was commenced to check for consistency and reliability of the questionnaire. Patients were interviewed to obtain sociodemographic data. Relevant medical history and laboratory parameters were obtained from patients' records. One supervisor (MD+) and two data collectors (MD) participated in the data collection process. Quality of data was ensured through training and supervision of data collectors.

### 2.8. Data Analysis

Data were entered into EPI Info version 4.4.1 and transported to SPSS version 20 for analysis.

Patient characteristics were reported as counts (percentages) for categorical variables and mean with standard deviation for continuous variables. Bivariate and multivariate logistic regression models were constructed to identify associated factors with anticoagulation outcome. Crude odds ratio (COR) and adjusted odds ratio (AOR) were reported. A *P* value <0.05 was used to declare a significant association.

### 2.9. Ethical Considerations

The research protocol complied with the Declaration of Helsinki, and ethical clearance was obtained from the Institutional Review Board (IRB) of College of Medicine and Health Sciences, University of Gondar (04/09/2012; IRB No. 09/20/627/12). Study subjects were recruited only after informed written consent was obtained. All data obtained were treated confidentially. During the data collection process, those patients who were found to have atrial fibrillation and venous thromboembolism were taken care of as per the recommendations of AHA/ACC guidelines and American Society of Hematology guideline, respectively [2, 3].

### 2.10. Definition of Terms

The proportion of INRs within the desired range of 2.0–3.0 (2.5–3.5 for mechanical prosthetic valves) is defined as the number of INRs within the target range divided by the number of INR measurements per patient [22].

The proportion of time spent within the therapeutic INR range (TTR) is defined as the duration of time for which the patient's international normalized ratio (INR) values were within a therapeutic range of 2.0–3.0 (2.5–3.5 for mechanical prosthetic valves). It was calculated using Rosendaal's method, which has used linear interpolation to assign an INR value to each day between successive observed INR values. TTR was calculated as the number of person-days with therapeutic INR range divided by the total number of person-days on warfarin. TTR ≥ 65% declares “good” INR control, while TTR < 65% affirms “poor” INR control [23].

Warfarin adherence: “continuous, single interval measure of medication gaps” was used to assess medication refill for warfarin. It was calculated as the sum of the days a patient was late for warfarin pick-up appointments in each month of the year, divided by the total number of days between pick-up periods in the year of study. Nonadherence was defined as more than one-third of days late for warfarin pick-up appointments [24].

Major bleeding in nonsurgical patients is defined as fatal bleeding; and/or symptomatic bleeding in a critical area or organ, such as intracranial, intraspinal, intraocular, retroperitoneal, intra-articular or pericardial, or intramuscular with compartment syndrome; and/or bleeding causing a fall in hemoglobin of 2 gm/dl or more, or leading to transfusion of 2 or more units of whole blood or red cells [25].

Clinically relevant nonmajor bleeding in nonsurgical patients is defined as any sign or symptom of hemorrhage (e.g., more bleeding than would be expected for a clinical circumstance, including bleeding found by imaging alone) that does not fit the criteria for the ISTH definition of major bleeding but does meet at least one of the following criteria: requiring medical intervention by a healthcare professional; leading to hospitalization or increased level of care; prompting a face-to-face (i.e., not just a telephone or electronic communication) evaluation [26].

## 3. Results

### 3.1. Sociodemographic Characteristics of Study Participants

A total of 338 patients on warfarin were included in the study. The mean age of study subjects was 48.8 (SD = 16.4) years. The majority of study participants were females (217/338, 64%), married (247/338, 73%), and urban dwellers (193/338, 57%). Most respondents were Christian by religion (290/338, 86%), and half (164/338, 49%) of them attended formal education (Table 1).

### 3.2. Clinical Characteristics of Study Participants

**Figure 1:**
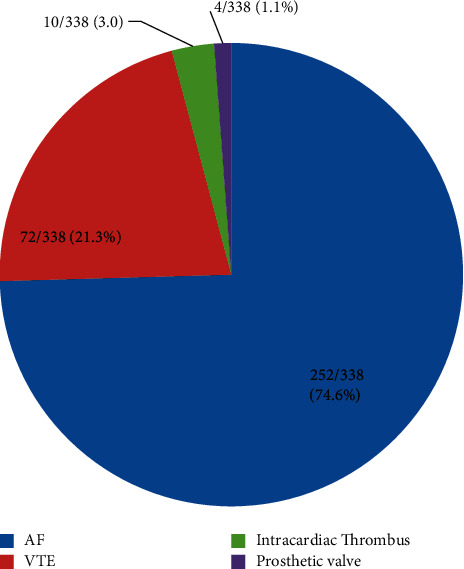
Pie chart showing number (percentage) of indications for warfarin therapy. AF, atrial fibrillation; VTE, venous thromboembolism.

Three-quarters (252/338, 75%) of patients received warfarin for atrial fibrillation (Figure 1). More than half (199/338, 59%) of patients were taking warfarin dose <5 mg, PO daily. Most patients (305/338, 90%) received other concomitant drugs like antihypertensive drugs (102/338, 30%), antiplatelets (144/338, 43%), lipid-lowering drugs (statins) (75/338, 22%), antithyroid drugs (PTU) (51/338, 15%), and antiretroviral drugs (ART) (22/338, 7%). Most patients (283/338, 84%) had INR determination every month. Heart failure and hyperthyroidism were detected in 144/338 (43%) and 46/338 (15%) patients, respectively. Most patients (277/338, 82%) never touched alcohol (Table 2).

### 3.3. Proportion of INRs within the Desired Range

Among 334 patients with atrial fibrillation, venous thromboembolism, and intracardiac thrombus, the proportion of desired INRs was determined. One-third (110/334, 33%) of patients achieved therapeutic INR range (INR = 2.0–3.0). However, half (165/334, 49%) of the patients achieved subtherapeutic INR range (INR < 2.0). The supratherapeutic INR range (INR > 3.0) was attained by less than one-fifth (59/334, 18%) of patients. Among 4 patients with mechanical prosthetic valves, only one patient (25%) attained the desired INR range (INR = 2.5–3.5), while the remaining, 3/4 (75%), achieved the subtherapeutic INR range (INR < 2.5).

### 3.4. Proportion of Time Spent in the Therapeutic INR Ranges

**Figure 2:**
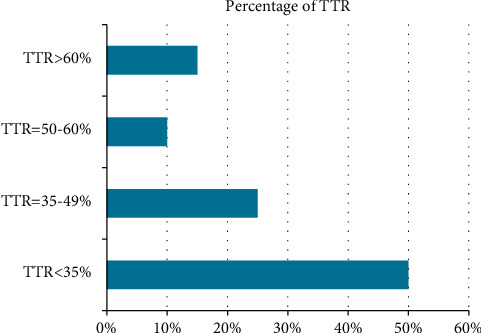
Bar diagram showing the percentage of TTR among patients on warfarin.

The proportion of time spent within the therapeutic range (TTR) was determined for all study participants (Figure 2). More than one-tenth (44/338, 13%) of patients achieved good INR control (TTR ≥ 65%). More than one-third (125/338, 37%) of study subjects had poor TTR control (TTR = 36–65%). Half (169/338) of the study subjects attained TTR < 35%, which was interpreted as no survival benefit from warfarin therapy.

### 3.5. Bleeding Events among Patients on Warfarin

A quarter (85/338, 25%) of patients on warfarin experienced clinically relevant nonmajor bleeding; this had required weekly INR determination and dose adjustment. Among patients with nonmajor bleeding episodes, almost all (77/85, 90%) had TTR < 65% and the remaining (8/85, 10%) had TTR ≥ 65%. No major bleeding episodes or vascular thrombosis were noticed among patients on warfarin.

### 3.6. Factors Associated with Anticoagulation Control

On bivariate analysis, VTE as indication for warfarin; warfarin dose, 5 mg PO daily; and absence of heart failure were predictors of good INR outcome (TTR ≥ 65%). None of the above variables were found to be significant when regressed on multivariate analysis. Bivariate analysis revealed no significant association of sociodemographic characteristics including age, gender, educational level, and monthly income with good INR outcome (TTR ≥ 65%). Similarly, clinical characteristics including dose of warfarin, warfarin adherence, INR monitoring interval, other concomitant drugs intake, other comorbidities, body mass index, and alcohol intake showed no significant association with good INR outcome (TTR ≥ 65%) (Table 3).

## 4. Discussion

Atrial fibrillation (75%) was the commonest indication for warfarin use, followed by venous thromboembolism and prosthetic heart valves. This finding was shared by reports from other sub-Saharan African countries [12–16]. The proportion of adequate anticoagulation outcome (TTR ≥ 65%) in the study subjects was 13%. The finding was on the lower margin of previous reports in sub-Saharan Africa, where the proportion of adequate INR level was 15–25% [12–14]. However, the United States and European studies have shown that the proportion of adequate anticoagulation level was 50–70% [7–11]. The reason for varied magnitude of anticoagulation control among countries was partly explained by their difference in anticoagulation management services. Reports from the Western world had shown that establishing anticoagulation clinics, implementing computer-assisted warfarin dosing, self-testing and self-management among motivated patients, and instituting coordinated follow-up clinics had improved the anticoagulation control [27, 28]. In this study, only one-third (33%) of study subjects attained the desired INR value (INR = 2.0–3.0) and majority (59%) were on low-dose warfarin (<5 mg, daily). Physicians might tend to undertreat patients because of fear of bleeding [29]. A quarter (24%) of patients were nonadherent to their medication. Patients' comprehension on health benefits of the oral anticoagulant might be low [30]. There was no validated warfarin-dose titration protocol in the hospital. Warfarin-dose titration was based on conventional clinical practice. “Anticoagulation clinic” for patients' close follow-up was not available. These challenging conditions might have contributed to poor anticoagulation control. On bivariate analysis, VTE as indication for warfarin; warfarin dose, 5 mg PO daily; and absence of heart failure were predictors of good INR outcome (TTR ≥ 65%). Patients with VTE might not have pill fatigue, since time to treatment was limited. Even though patients on higher warfarin dosage might achieve an acceptable INR target, variant alleles of the CYP2C9 and VKORC1 genes among patients might affect the warfarin-dose requirements, sensitivity to warfarin, and quality of anticoagulation. Few studies documented the presence of genetic polymorphisms to warfarin among African descents [31]. Patients with no heart failure might not be on multiple drugs, which then limited drug-drug interactions. On multivariate analysis, explanatory sociodemographic and clinical characteristics showed no significant association with good TTR outcome (TTR ≥ 65%). Global studies documented that old age, obese individuals, other concomitant drugs intake, excessive alcohol intake, and renal or hepatic dysfunctions were among the listed causes of poor TTR outcome (TTR < 65%) [8–11, 17–21]. Direct oral anticoagulants (DOACs) are recently introduced anticoagulants, which have a number of advantages over warfarin, despite limited access, cost issues, and availability of antidote. DOACs are prescribed in fixed doses, have fewer interactions with food and drugs, and do not require routine anticoagulant monitoring. Their use might be considered for eligible patients with atrial fibrillation and venous thromboembolism [9–11, 14, 16].

### 4.1. Limitation of the Study

Since it was a cross-sectional study, the determined anticoagulation level might not be a true reflection of what happens all the time. Generalizability to the study population was limited, since the nonprobability sampling method was used to recruit study subjects.

## 5. Conclusion

The quality of anticoagulation control with warfarin in the study subjects was very low. The authors recommend to implement a validated warfarin-dose titration protocol and to establish anticoagulation clinics to mitigate the low anticoagulation level.

## Figures and Tables

**Table 1:** Sociodemographic characteristics of patients taking warfarin in University of Gondar hospital, Northwest Ethiopia, from November 1, 2019, to October 31, 2020 (*n* = 338).

Variables	Frequency (no.)	Percentage
Age (years)		
<40 years	115	34.0
40–60 years	110	32.5
>60 years	113	33.5
Gender		
Male	121	35.8
Female	217	64.2
Residence		
Urban	193	57.1
Rural	145	42.9
Educational level		
Cannot read and write	71	21.0
Informal education	103	30.5
Elementary school	51	15.1
Secondary school	64	18.9
College and above	49	14.5
Monthly income (in birr)		
<1500	183	54.1
1500–3000	85	25.1
>3000	70	20.8

**Table 2:** Clinical characteristics of patients taking warfarin in University of Gondar hospital, Northwest Ethiopia, from November 1, 2019, to October 31, 2020 (*n* = 338).

Clinical characteristics	Frequency (no.)	Percentage
BMI (kg/m^2^)		
<18.5	29	8.6
18.5–24.9	229	67.7
25.0–29.9	53	15.7
≥30	27	8.0
Indication for warfarin		
Atrial fibrillation	252	74.6
Venous thromboembolism	72	21.3
Prosthetic heart valves	4	1.1
Intracardiac thrombus	10	3.0
Dose of warfarin		
<5 mg, daily	199	58.9
5 mg, daily	118	34.9
>5 mg, daily	21	6.2
INR monitoring interval		
Every 2 weeks	32	9.5
Every 1 month	283	83.7
Every 2 months	23	6.8
Nonadherence to warfarin		
Yes	82	24.3
Concomitant drugs use		
Yes	305	90.2

BMI, body mass index; concomitant drugs use included antiplatelets, statins, antihypertensives, antithyroid drugs, and antiretroviral drugs.

**Table 3:** Bivariate and multivariate logistic regression analyses of time spent within the therapeutic INR range (TTR) among patients taking warfarin in University of Gondar, Northwest Ethiopia, from November 1, 2019, to October 31, 2020 (*n* = 338).

Variables	TTR		COR (CI)	*P* value	AOR (CI)	*P* value
	TTR < 65%	TTR ≥ 65%				
Age						
<40 years	100	15	1.04 (0.48, 2.27)	0.922		
40–60 years	97	13	0.94 (0.42, 2.12)	0.877		
>60 years	99	14	1			
Gender						
Male	103	18	1			
Female	193	24	1.41 (0.73, 2.71)	0.310		
Marital status						
Single	50	11	1			
Married	218	28	1.98 (0.51, 7.71)	0.325		
Others	27	3	1.15 (0.33, 4.04)	0.825		
Monthly income						
<1500 birr	157	26	1			
1500–3000 birr	77	8	1.28 (0.55, 2.99)	0.563		
>3000 birr	62	8	0.81 (0.29, 2.27)	0.682		
Educational level						
Cannot read and write	61	10	1			
Informal education	91	12	1.80 (0.53, 6.12)	0.345		
Elementary school	45	6	1.45 (0.44, 4.76)	0.539		
Secondary school	54	10	1.47 (0.39, 5.56)	0.573		
College and above	44	4	2.04 (0.60, 6.94)	0.255		
Residence						
Urban	170	23	0.89 (0.47, 1.72)	0.744		
Rural	126	19	1			
BMI (kg/m^2^)						
<18.5	25	4	1			
18.5–24.9	199	30	1.44 (0.39, 5.19)	0.578		
≥25	72	8	1.36 (0.59, 3.09)	0.469		
Indication for warfarin						
Atrial fibrillation	222	30	1			
VTE	64	8	0.14 (0.02, 0.99)	0.049	0.56 (0.14, 2.18)	0.404
Others	10	4	0.31 (0.08, 1.23)	0.097	0.43 (0.09, 1.94)	0.271
Dose of warfarin						
<5 mg daily	181	18	1			
5 mg daily	98	20	0.39 (0.12, 1.32)	0.133	0.40 (0.09, 1.64)	0.282
>5 mg daily	17	4	0.91 (0.28, 2.98)	0.877	0.94 (0.24, 3.74)	0.950
Nonadherence to warfarin						
Yes	40	2	1			
No	266	30	2.26 (0.52, 9.81)	0.278		
Concomitant drugs use						
Yes	268	37	1			
No	28	5	1,29 (0.47, 3.56)	0.618		
Frequency of INR monitoring						
Every 2 weeks	28	4	0.68 (0.15, 3.05)	0.613		
Every 1 month	249	34	0.65 (0.21, 2.02)	0.455		
Every 2 months	19	4	1			
Alcohol intake						
Yes	54	6	1			
No	241	36	1.34 (0.54, 3.34)	0.531		
Chronic liver disease						
Yes	14	3	0.91 (0.46, 1.80)	0.791		
No	122	16	1			
Unknown	160	23	1.49 (0.39, 5.59)	0.554		
Chronic kidney disease						
Yes	25	4	1.32 (0.62, 2.85)	0.473		
No	184	28	1			
Unknown	87	10	1.39 (0.40, 4.82)	0.602		
Heart failure						
Yes	130	14	1			
No	166	28	0.64 (0.32, 1.26)	0.197	1.70 (0.80, 3.61)	0.168
Hyperthyroidism						
Yes	45	6	1			
No	251	36	0.93 (0.37, 2.33)	0.877		

BMI, body mass index; VTE, venous thromboembolism; concomitant drugs use included antiplatelets, statins, antihypertensives, antithyroid drugs, and antiretroviral drugs.

## Data Availability

All data generated and analyzed are included within this research article.

## Abbreviations

ACC: American College of Cardiology
AHA: American Heart Association
AOR: Adjusted odds ratio
ART: Antiretroviral therapy
CI: Confidence interval
COR: Crude odds ratio
DOACs: Direct oral anticoagulants
INR: International normalized ratio
IRB: Institutional Review Board
PTU: Propylthiouracil
TTR: Time spent within the therapeutic range
VTE: Venous thromboembolism.
